# A carrier-free long-acting ropivacaine formulation using methylprednisolone sodium succinate as a dual-functional adjuvant

**DOI:** 10.7150/thno.107397

**Published:** 2025-03-03

**Authors:** Weiwei Wu, Yan Wang, Feng Qiu, Yingxian Dong, Jing Liu, Yujun Zhang, Deying Gong, Yi Kang, Guoyan Zhao, Congyan Liu, Yuncheng Li, Tao Zhu, Wensheng Zhang, Guowei Che

**Affiliations:** 1 Department of Anaesthesiology, West China Hospital, Sichuan University, Chengdu, China.; 2 Department of Thoracic Surgery, West China Hospital, Sichuan University, Chengdu, China.; 3 Laboratory of Anaesthesia and Critical Care Medicine, National-Local Joint Engineering Research Centre of Translational Medicine of Anaesthesiology, West China Hospital, Sichuan university, Chengdu, China.; 4 Research Unit for Perioperative Stress Assessment and Clinical Decision, Chinese Academy of Medical Sciences (2018RU012), West China Hospital, Sichuan University, Chengdu, China.; 5 Lung Cancer Center, West China Hospital, Sichuan University, Chengdu, China.

**Keywords:** long-acting analgesia, carrier-free formulation, pure drug self-assembly, drug microcrystals, slow release

## Abstract

**Rationale:** Slow-releasing formulation of local anesthetics (LAs) has been a promising strategy for pursuing long-acting opioid-free analgesia. Although many formulations based on carrier materials have been successfully developed, challenges still remain in addressing not only the biocompatibility and biodegradability issues of carrier materials, but also potential local inflammation caused by LAs.

**Methods:** In this study, we developed a slow-releasing ropivacaine formulation based on methylprednisolone sodium succinate (MP), a small-molecule drug used in clinic. Firstly, we studied the self-assembling behavior of MP and its interaction with ropivacaine hydrochloride (RH). Then we studied how MP could manipulate the crystallization of RH and how the release profile of obtained ropivacaine crystals could be controlled. Lastly, we investigated the long-acting analgesic effect and safety of different formulations in animal models. Meanwhile we also monitored the anti-inflammatory effect of MP on cell and animal levels.

**Results:** MP could self-assemble into nanoparticles, which could adsorb RH and induce the formation of homogeneous ropivacaine microcrystals. Higher MP ratio in the system led to the formation of smaller ropivacaine microcrystals with a moderate release rate, which generated much longer and reliable analgesic effect in animal models with considerable safety. On the other hand, MP in the formulation showed substantial anti-inflammatory effect, which was also helpful to further relieve pain and alleviate local toxicity.

**Conclusion:** Using MP as a dual-functional adjuvant, long-acting LA formulations with considerable safety could be prepared, providing a facile solution for long-term pain management in clinic.

## Introduction

The management of postoperative pain has long been one of the most important issues in clinical practice 1. Compared with opioids, local anesthetics (LAs) have fewer side effects and are non-addictive, encouraging their wide application in relieving postoperative pain 2-4. However, the effective duration of traditional LAs is limited to 6-8 h, which is far from meeting the demand of long-term analgesia for postoperative pain generally lasting for 48-72 h 5.

Novel carrier materials have shown great advantages in pharmacological research by affecting the dissolving, releasing, distribution and clearance of drugs 6-9. In order to improve the analgesic effect of LAs in postoperative pain management, a common strategy is to develop their slow-releasing formulations using carrier materials. In recent years, various materials including microspheres, gels, and liposomes have been developed for the slow-release of LAs 10-13. Among them, liposomes appear to be the most promising carrier material. EXPAREL, the first long-acting LA formulation approved by the FDA, represents a significant advancement in this field and has shown its promising advantage in clinical application 14-16. However, using carrier materials also brings potential problems such as complicated and costly production process, low drug loading capacity, concern of formulation stability, and so on. For these reasons, long-acting LA formulations containing novel materials usually need a long way to be approved for clinical application, and only a few have been successfully brought to the clinic stage 17-19. Furthermore, among the currently available formulations, Zynrelef and POSIMIR are gels with low injectability, while Xaracoll is an implant and cannot be injected, thus limiting their use to a few clinical scenarios only. Additionally, some post-marketing clinical studies on EXPAREL have yielded results that are less than satisfactory 20.

On the other hand, it is known that the anesthetic effect of LAs is also inevitably accompanied by mild local and systemic toxicity 21, 22. Although their effects are generally recoverable, transient, and clinically acceptable, such conditions have set limits on the concentration and dose of LAs allowed. Even though slow-releasing formulations can effectively control the local and plasma drug concentration to avoid lethal acute toxicity, long-term exposure of local tissue to low-concentration LAs seems also problematic. For example, notable local inflammation has been reported for some long-acting LA formulations as the anesthetic duration is extended 23, 24. More and more studies have indicated the importance of adding anti-inflammatory ingredients in the formulation while pursuing long-acting analgesia 25, 26.

**Figure 1:**
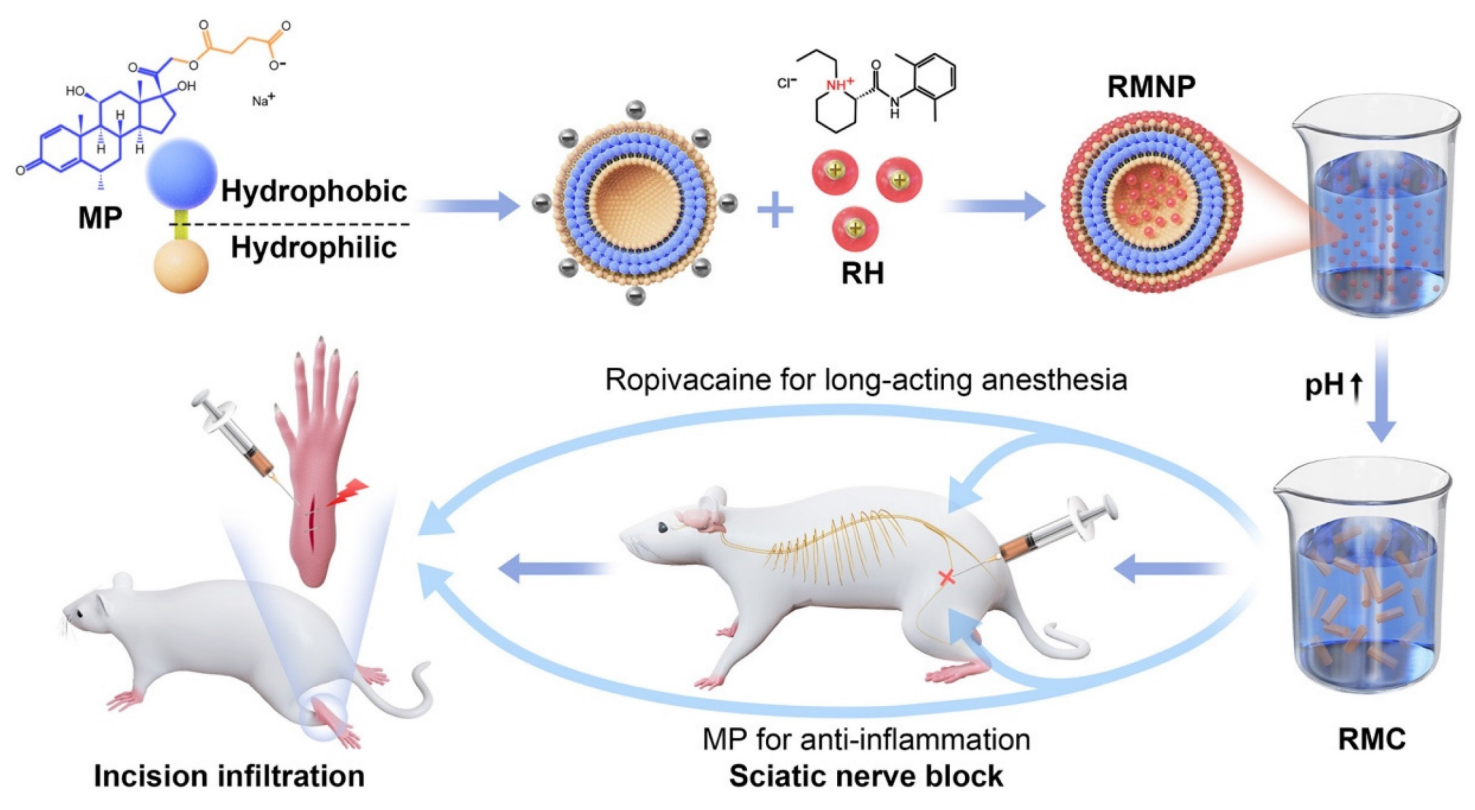
Schematic illustration of the self-assembly of MP and its application as a dual-functional adjuvant for the preparation of long-acting RMC formulation.

In this study we introduced a carrier-free ropivacaine formulation using methylprednisolone sodium succinate (MP), an inflammatory glucocorticoid as a dual-functional adjuvant. We found that MP could self-assemble into nanoparticles in aqueous solution, which could adsorb ropivacaine hydrochloride (RH) to form ropivacaine-MP nanoparticles (RMNPs). Based on RMNPs pre-formed in the solution, crystallization of RH could be induced by increasing pH, resulting in a slow-releasing suspension containing ropivacaine-MP microcrystals (RMCs). By adjusting the ratio between RH and MP to control the size of RMCs, the release rate of ropivacaine could be well-controlled, leading to an optimized analgesic effect in animal models. On the other hand, MP in the formulation also showed anti-inflammatory activity and considerably alleviated local tissue damage (Figure 1).

## Materials and Methods

### Materials

RH (> 98% purity) was purchased from Aladdin (Shanghai, CN). MP (95% purity) was purchased from APExBIO Technology LLC (Houston, US). All other chemicals were of analytical purity and available commercially. Bupivacaine liposome (Bupi-Lipo) was purchased from Jiangsu Hengrui Medicine Co., Ltd (Lianyungang, Jiangsu, CN).

### Preparation of MP nanoparticles, RMNPs and RMCs

MP was dissolved in Milli-Q water at the desired concentration as described in the following sections to study its self-assembling behavior. To prepare RMNPs, RH at a fixed concentration of 50 mg/mL was mixed with MP at different RH:MP molar ratios ranging from 15:1 to 75:1. The original pH of RH, MP and RMNP was around 5.5, 6.5 and 6.0, respectively. Complex solutions containing RMNP-L (lowest MP proportion, RH:MP molar ratio = 75:1) or RMNP-H (highest MP proportion, RH:MP molar ratio = 15:1) were used to prepare RMCs. Briefly, the pH of RMNP-L and RMNP-H was adjusted to 8.0 under vigorous stirring (300 rpm) to obtain suspension of RMC-L and RMC-H, respectively. As a comparison, ropivacaine crystal (RC) was also prepared by adjusting the pH of RH solution (50 mg/mL) to 8.0 using the same protocol. All crystal formulations were stored at -4°C as lyophilized powder and resuspended with PBS to desired concentration before use. To simplify the comparisons, the concentration defining different formulations was the total amount of RH in each formulation. All samples were prepared and investigated at room temperature unless specified elsewhere.

### Characterization of MP nanoparticles, RMNPs and RMCs

#### Atomic force microscopy (AFM)

The morphology of MP, RMNP-L and RMNP-H nanoparticles was observed by using AFM. Briefly, 10 μL of each sample was dropped onto the surface of freshly-cleaved mica and air-dried. The self-assembling structures of MP and RMNPs were observed using AFM (Shimadzu, JP), and the height of nanostructures was analyzed using the line profile analysis tool provided in the AFM software.

#### Dynamic light scattering (DLS)

Particle size of MP, RMNP-L and RMNP-H nanoparticles used for AFM study was measured by using a ZS90 particle size analyzer (Malvern Instruments Ltd., UK). For RMNPs with different RH:MP molar ratios, the zeta potential was also measured. The particle size and specific surface area of RMCs were analyzed by a Mastersizer 3000 laser particle size analyzer (Malvern Instruments Ltd., UK). Briefly, RMCs were dispersed in 500 mL of saturated solutions, the shading was adjusted to 8-20% and each sample was tested three times with stirring.

#### Measurement of pyrene fluorescence

To measure the critical aggregation concentration (CAC) of MP at different pH, solution of MP with an initial pH of 6.5, or adjusted to 6.0 (by 0.1 mol/L HCl) and 7.5 (by 1 mol/L NaOH), were prepared at concentrations ranging from 0.03125 mg/mL to 8 mg/mL. Then pyrene stock solution (2 mmol/L in DMSO) was added into different MP solutions to reach a final pyrene concentration of 4 μmol/L, and pyrene fluorescence was measured by a spectra fluorophotometer (Horiba Ltd., JP) with excitation wavelength of 336 nm. The ratio between the fluorescent intensity at 370 nm (I1) and 380 nm (I3) was plotted against MP concentration, and the concentration where the ratio began to drop quickly was defined as the CAC value. To investigate the impact of solvent polarity on the CAC of MP, tetrahydrofuran (THF) solutions with different concentrations ranging from 10% to 40% were used to prepare MP solutions with concentrations ranging from 0.03125 to 8 mg/mL. Then the CAC values of MP in different THF solutions were determined by the pyrene fluorescence method as described above.

#### Scanning electron microscopy (SEM)

The morphology of RMC and RC was examined using SEM. Samples were spread on a clean glass slide, allowed to dry and then coated with gold using a 108 Auto sputter coater (Cressington Scientific Instruments Ltd., UK). SEM images were then collected using an EVO 10 scanning electron microscope (Zeiss, DE).

#### X-Ray diffractometer (XRD) and Fourier transform infrared (FTIR)

The crystal structures of lyophilized RMC, RC, RH and MP were determined using X-Ray diffractometer (PANalyticalB.V, NL). Diffractograms were run in scanning steps of 0.02°, between 2θ values (5-50°). The FTIR spectra of different samples were analyzed using an INVENIO R spectrometer (BRUKER, DE).

### *In vitro* ropivacaine release

Release experiment was carried out using dialysis tubes to investigate the *in vitro* release profile of different formulations including RH, RC, RMNP-L, RMNP-H, RMC-L and RMC-H. Briefly, 500 μL of each formulation with the concentration equal to 5% RH was added into a dialysis tube with 10 kD molecular weight cut-off (Spectrum labs, USA), which was dipped into 40 mL of PBS (pH 7.2) and incubated at 37°C with continuous stirring. To prevent the released ropivacaine from getting saturated in PBS, a half volume of each sample was taken out at 0.5, 1, 2, 4, 8, 12 and 24 h, and then once every other 24 h, and 20 mL of fresh PBS was added after each sampling. The amount of released ropivacaine from each formulation was measured by high performance liquid chromatography as described previously 27.

### Animals

Adult male Sprague-Dawley rats weighing 250-300g were obtained from Dossy Experimental Animal Co., Ltd. (Chengdu, CN) and housed in groups in a 7 a.m.-7 p.m. light-dark cycle. Animals were cared for in accordance with protocols approved by the Animal Ethical Committee of West China Hospital, Sichuan University (approval number 2020018A), and all animal experiments were carried out following the National Institutes of Health Guide for Care and Use of Laboratory Animals.

### Pharmacokinetics study

All rats were randomly assigned to experimental groups based on body weight (n = 8). To minimize potential experimental bias, the study employed a double-blind design: drug administration and outcome assessment were conducted by separate personnel. Different formulations including 1% RH, 5% RH, 5% RMC-L and 5% RMC-H were respectively injected into the sciatic nerve area of rats, and then 0.2 mL of blood samples were collected from the tail vein at predetermined time points including before administration and 0.17, 0.5, 1, 2, 4, 5, 8, 12, 14, 24, 28, 32, 48, 52, 56 and 72 h post-administration. Then the plasma was separated by centrifugation at 3500 rpm for 10 minutes and 50 μL of plasma samples were added to 150 μL of acetonitrile containing internal standard (ropivacaine-d7: 10 ng/mL), before being vortexed and centrifuged at 20,000 rpm for 10 min at 4°C. The supernatant was collected for high performance liquid chromatography-mass spectrometry (Agilent 1260-6460, Agilent Technology, US) measurement to determine the plasma drug concentration as described previously 27. The results of pharmacokinetics study were analyzed using Drug and Statistic Version 3.0 (DAS 3.0, the Mathematical Pharmacology Committee, Chinese Pharmacological Society, CN).

### Rat sciatic nerve block (SNB) model

The randomization and blinding procedures for the SNB experiment were the same as described above in section 2.6 (n = 8). Rats were anesthetized with sevoflurane and shaved to expose the skin of the left thigh, then 0.2 mL of normal saline (NS), RH, Bupi-Lipo or RMC at different concentrations and RH:MP ratios were injected into the left sciatic nerve area using a 26G needle. The classic hot plate test was used to evaluate the sensory block by measuring paw withdrawl latency (PWL) 28. Briefly, the left hind paw was placed on a hot plate (55°C) and the duration until the paw withdrawal was recorded, and a cutoff value of 12 s was set to avoid burns 29. Effective block was calculated as percentage of maximum possible effect (%MPE).



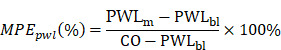



PWL_m_: the measured value of PWL; PWL_bl_: the baseline of PWL; CO: the cutoff value of 12 s.

To evaluate motor block by measuring the postural extensor thrust (PET), the rat was lifted vertically and the left hind limb was stomped on the electronic scale to read the thrust force.

For each rat, the baselines of PWL and PET were measured before drug administration, and then the PWL and PET were monitored at time points including 0.17, 2, 4, 6, 8, 10, 12, 24, 28 and 32 h after administration until the value returned to the baseline.

### Rat footpad incisional pain model

The randomization and blinding procedures for the incisional pain model were the same as described above in section 2.6 (n = 8). Rats were anesthetized by sevoflurane, and an incision of approximately 1 cm long was made in the metatarsal region of the left foot, with the incision approximately 0.5 cm from the heel. The plantaris muscle was incised longitudinally, keeping the muscle origin and insertion site intact. The wound was closed using 5-0 nylon sutures. A 26G needle was used to inject 0.1 mL of NS, 1% RH, 5% RMC-L or 5% RMC-H around the wound. The mechanical response threshold (MRT) of each rat was monitored at predetermined time points: before surgery (baseline) and 0.5, 2, 4, 6, 8, 24, 48 and 72 h after surgery and drug administration. For MRT measurement, the rat was placed in a transparent plastic cage (21* 27* 15 cm^3^) on an elevated plastic grid plate (8 x 8 mm^2^). The skin around the plantar incision was stimulated using an electronic Von frey (Bioseb, FR) to read the value causing a withdrawal response. For each rat at each time point, an averaged value was calculated from three reads.

### Histological analysis

The rats in the SNB model were euthanized on day 4 and 14 after drug injection (n = 4 for each time point), and sciatic nerve and surrounding muscle were harvested. Tissue specimen slides were stained with hematoxylin and eosin (HE), following which sciatic nerve inflammation, axonal degeneration, myocyte inflammation and myotoxicity were scored. For rats euthanized on day 14, heart, liver, spleen, lung and kidney were also harvested for HE staining.

Similarly, the rats in the incisional pain model were euthanized on day 7 and 14 after drug injection (n = 4 for each time point), and the skin and subcutaneous tissue were collected from the incision sites. The tissue specimen slides were stained with HE, following which inflammatory infiltration and the degree of granulation tissue proliferation at the incision site were evaluated.

### Immunofluorescence assay of dorsal root ganglion (DRG)

The rats in the incisional pain model were euthanized on day 7 following the drug injection, and DRG of 4, 5 lumbar segments (L4, L5) were harvested and post-fixed overnight (n = 3). After sucrose gradient dehydration, DRG was sectioned into slices with 4 μm thickness. TRPV1 and c-Fos in DRG were stained using the TRPV1 kit (Affinity, DF6378) and the c-Fos kit (Servicebio, GB12096) following the manufacturing instructions. The tissue sections were deparaffinized, subjected to antigen retrieval, and treated to inhibit endogenous peroxidase activity, followed by antigen blocking. Primary antibodies against TRPV1 and c-Fos (1:200 dilution) were applied, and the sections were incubated overnight at 4°C. After washing, secondary antibodies were applied and incubated at room temperature for 50 minutes in the dark. Nuclear counterstaining was performed using DAPI staining solution (Servicebio, G1012) for 10 minutes at room temperature. After washing, autofluorescence was suppressed using a quenching reagent, and the sections were mounted for microscopic analysis. Images were acquired using fluorescence microscopy (Nikon ECLIPSE Ti2). The number of positive neuron cells and mean fluorescence intensity (MFI) were calculated using Image J (2.14.0).

### Cytotoxicity assay

To investigate the protective effect of MP in the RMC formulation, C2C12 and PC12 cells were used to evaluate the cytotoxicity of RH, MP and RMC. Cells were seeded in 96-well plates at a density of 6*10^3^ cells per well and cultured for 24 h, with 4 replicates per group. Fresh mediums containing 1, 3 or 5 mmol/L of RH, or RMC with the same dose of RH were prepared. As comparisons, fresh medium containing MP at the concentration of 0.07, 0.2 or 0.3 mmol/L, which was respectively equal to the dose of MP in the RMC formulations, were also prepared. Cells were incubated with different drug-containing medium for another 24 h, after which cell viability was analyzed using an Enhanced cell counting kit-8 (CCK-8, Saint-Bio, China) following the manufacturer's instruction.

### Enzyme-linked immunosorbent assay (ELISA)

To evaluate the anti-inflammatory effect of MP in the RMC formulation, the levels of TNF-α, IL-1β and IL-6 around the sciatic nerve after drug injection were determined by ELISA. Formulations including NS, 1% RH, 1% RMC-H and 5% RMC-H were respectively injected around the sciatic nerve as described in section 2.7, then sciatic nerve and the surrounding muscle tissues were collected at 12 and 24 h after administration. Tissue homogenates were prepared, centrifuged and the supernatant was stored at -80°C before measurement. TNF-α, IL-1β and IL-6 levels were measured using the ELISA kit (Thermo Fisher Scientific Inc., US) following the manufacturer's instruction.

### Statistical methods

Continuous data were compared between groups using ANOVA if they followed a normal distribution, or the Kruskal-Wallis rank sum test if they did not obey a normal distribution. Categorical data were compared using the χ^2^ test or the Kruskal-Wallis rank sum test, depending on they were unordered or ordered. Two-by-two comparisons between multiple groups were performed using the Bonferroni correction. Data from repeated measures of the same dependent variable were compared using repeated measures ANOVA if Mauchly's test of sphericity was satisfied, or the Kruskal-Wallis rank sum test if not. All statistical analyses were conducted using RStudio (version 4.1.2, R Core Team, 2021, Vienna, Austria.).

## Results

### Self-assembly of MP and formation of RMNPs

**Figure 2:**
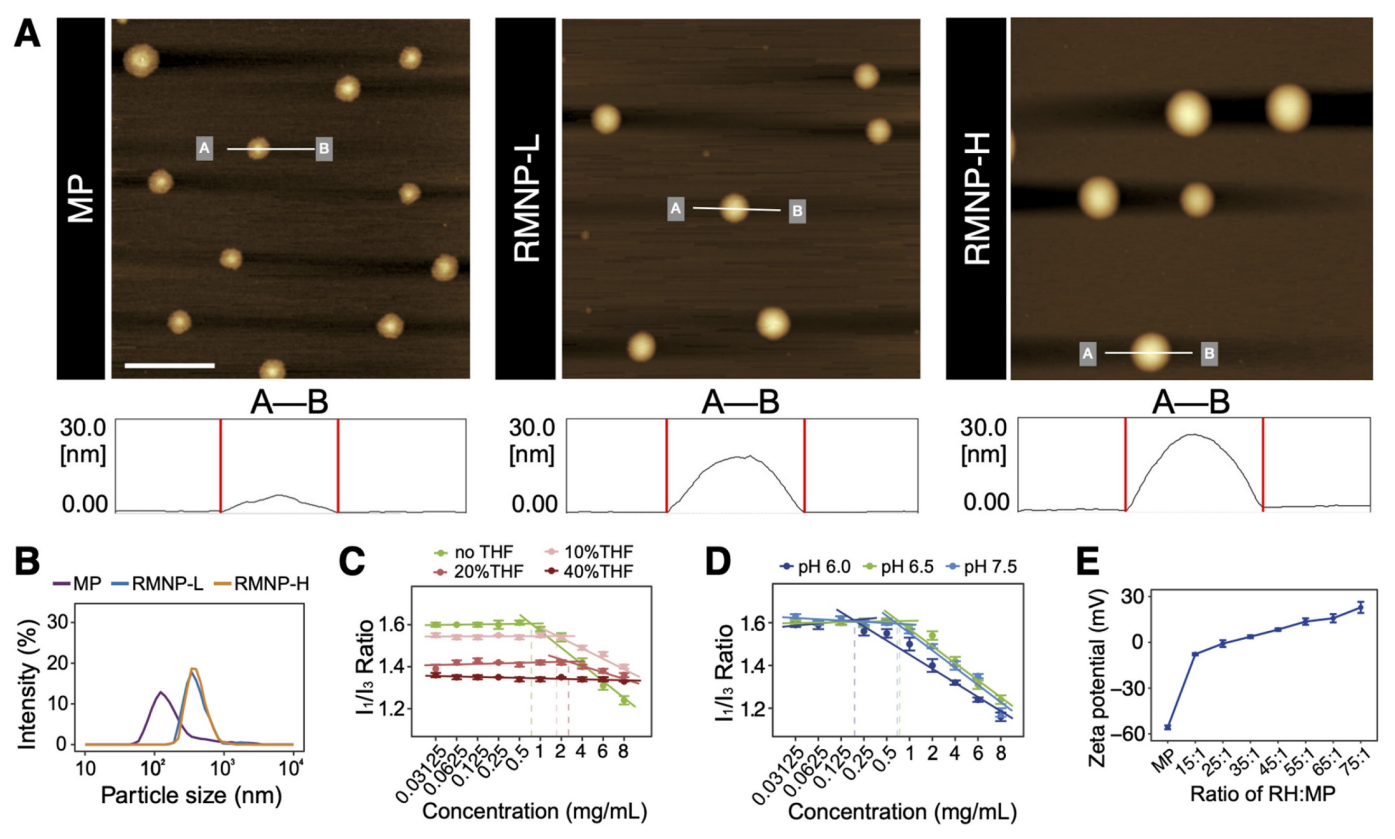
Characterization of MP nanoparticles and RMNPs. (A) AFM images of MP, RMNP-L and RMNP-H. Scale bar = 1 µm. Line profile analysis in the lower panel shows the height of different nanoparticles. (B) Representative particle size distribution of MP, RMNP-L and RMNP-H. (C) CAC value of MP in different concentration of THF revealed by the change of I_1_/I_3_ ratio of pyrene fluorescence. (D) CAC value of MP at different pH revealed by the change of I_1_/I_3_ ratio of pyrene fluorescence. (E) Zeta potential of MP and RMNP with different RH:MP ratio.

As shown if Figure 2A, MP could self-assemble into homogeneous nanoparticles in aqueous solution. On the contrary, RH as a highly soluble small molecule did not show any self-assembling behavior like MP (data not shown). When RH and MP were mixed at different ratios, nanoparticles of RMNP-L and RMNP-H with similar size were obtained. More interestingly, although the diameter of different particles revealed by AFM was similar, their height was quite different as shown by line profile analysis in the lower panel of Figure 2A. MP nanoparticles seemed to collapse to flat discs on the mica surface, probably due to their hollow inner space. On the other hand, RMNPs kept their solid spherical shape, suggesting the filling of RH into the inner space. As revealed by DLS, the mean particle diameters of MP, RMNP-H and RMNP-L were 166.6 ± 5.7 nm, 447.6 ± 14.0 nm and 535.9 ± 10.3 nm, respectively (Figure 2B). The size of RMNPs revealed by DLS was much bigger than that shown in AFM images, probably due to the aggregation of the nanoparticles.

As proposed in Figure 1, the methylprednisolone moiety of MP is hydrophobic while the succinate moiety with a negative charge is hydrophilic. Driven by hydrophobic interaction, this amphiphilic molecule could form liposome-like structure with negatively charged inner core and outer surface. To confirm this mechanism, we firstly used pyrene as an indicator to investigate the hydrophobic interaction. As shown in Figure S1, pyrene fluorescent spectrum of 4 mg/mL MP exhibited an obvious increase of the I_3_ peak as compared with the spectra of pure water or 40 mg/mL RH, suggesting that hydrophobic interaction was involved in driving the self-assembly of MP 30. As shown in Figure 2C, the I1/I3 value of MP dissolved in pure water began to drastically drop as the concentration reached between 0.5-1 mg/mL, which indicated that MP began to undergo self-assembly at concentration above this CAC value. While in 10% and 20% THF, the CAC value of MP increased to 1-2 mg/mL and 2-4 mg/mL respectively. In 40% THF, no CAC value was detected, suggesting that MP lost its ability to self-assemble. Since THF is an organic solvent destroying hydrophobic interaction, these results further confirmed that the self-assembly of MP greatly relied on hydrophobic interaction.

On the other hand, the pH of the water solution could also impact the self-assembly of MP. As shown in Figure 2D, the CAC value of MP kept almost unchanged when the pH was increased from 6.5 to 7.5, while at a lower pH of 6.0, the CAC value dropped to 0.125 - 0.25 mg/mL, which means MP was more prone to aggregate at lower pH. The impact of pH could be explained by the dissociation equilibrium and pKa of MP as shown in Figure S2, depending on which the charge state of MP could be roughly determined. At the pH of 6.5 and 7.5 which were much higher than the pKa value, the carboxyl group of MP was fully dissociated to bear strong negative charge. While at the pH of 6.0 which was closer to the pKa value, the negative charge became weaker so that MP could undergo self-assembly more readily due to the loss of strong intermolecular electrostatic repulsion. Such a mechanism of how pH change affecting the self-assembling behavior was very common in amphiphilic molecule systems, which further confirmed the self-assembling model of MP proposed in Figure 1.

As shown in Figure 2E, zeta potential of MP was -55.7 ± 1.3 mV, confirming the exposure of negatively charged carboxyl groups on the surface of nanoparticles. With the molar ratio of RH:MP rising from 15:1 to 75:1, zeta potential of RMNPs gradually increased from -4.07 ± 0.06 mV to 22.9 ± 3.65 mV, suggesting that positively charged RH molecules were adsorbed onto the surface of MP nanoparticles. The decrease of net surface charge caused by RH adsorbing could also explain the potential aggregation behavior of RNMPs as revealed by DLS. However, the RMNPs system based on electrostatic interaction was not an efficient slow-releasing formulation due to the high solubility of RH and low drug encapsulation efficiency (EE), which was only 12.64 ± 0.26% and 23.39 ± 0.43% for RMNP-L and RMNP-H, respectively (Table 1).

### Formation of slow releasing RMCs

**Figure 3:**
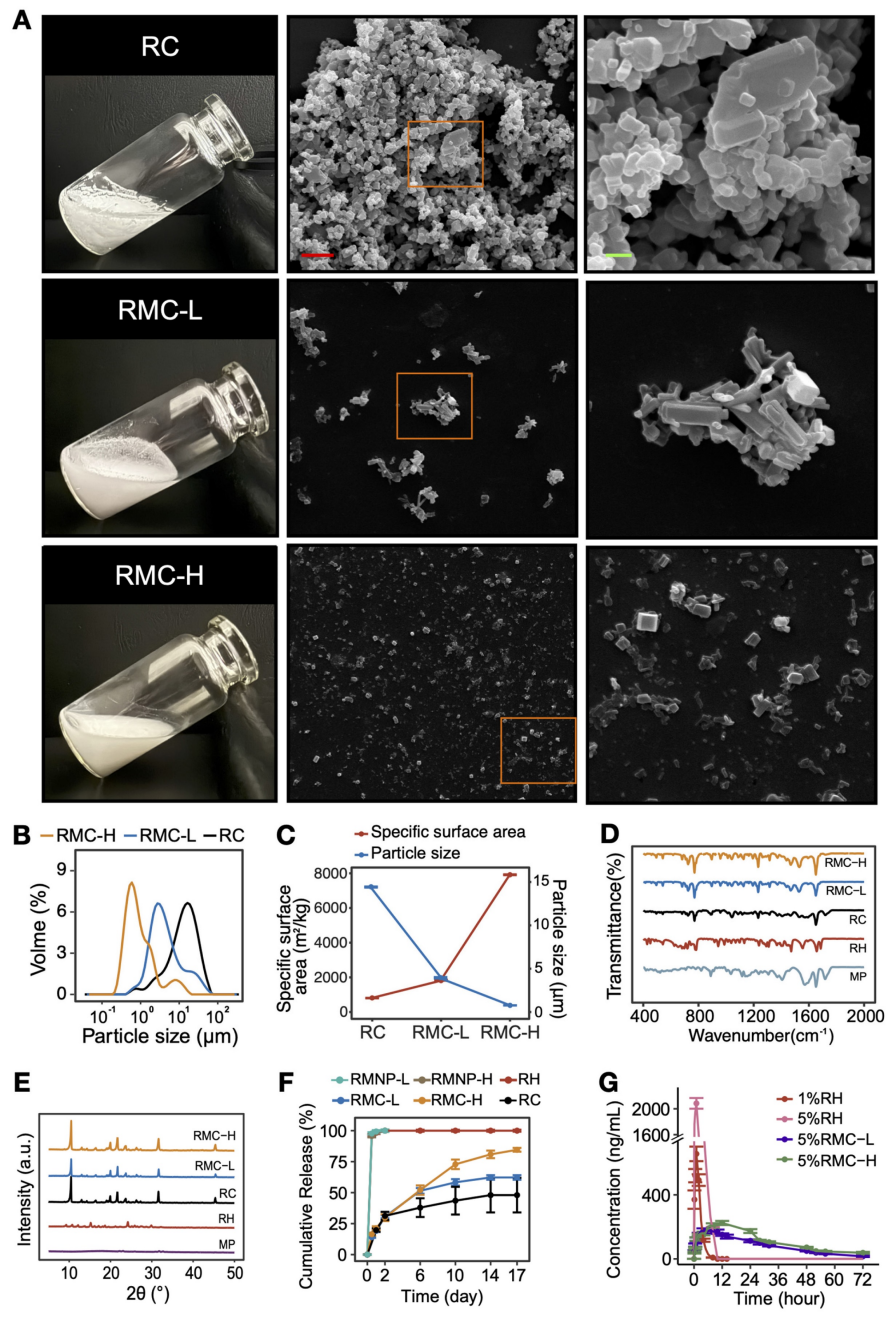
Characterization and slow-release profile of RMC. (A) Photo pictures and SEM images of RC, RMC-L and RMC-H. Right panel (scale bar = 2 µm) shows magnified images of the framed areas in the middle panel (scale bar = 10 µm). (B) Representative particle size distribution of RC, RMC-L and RMC-H. (C) Mean particle size and corresponding specific surface area of RC, RMC-L and RMC-H. (D) FTIR spectra of RC, RMPC-L, RMPC-H, RH and MP. (E) XRD spectra of RC, RMPC-L, RMPC-H, RH and MP. (F) *In vitro* release profile of RH, RMNP-L, RMNP-H, RMC-L, RMC-H and RC. All samples were at the same concentration of 5%. Data are shown as means ± standard error, n = 3. (G)* In vivo* release of 1% RH, 5% RH, 5%RMC-L and 5%RMC-H revealed by pharmacokinetic study. Data are shown as means ± standard error, n = 8.

It is known that soluble RH will transform to insoluble RC when pH rises to above its pKa value (Figure S2). However, raising the pH of pure RH led to the formation of large RC with a high tendency of aggregation, which could not be well dispersed in water (Figure 3A). On the other hand, when we increased the pH of the RMNP solutions to 8.0, much smaller and well-dispersed microcrystals were obtained (Figure 3A-B). As estimated by DLS, the mean particle size of RC was 14.57 ± 0.06 µm, while that of RMC-L and RMC-H was 4.02 ± 0.08 µm and 0.81 ± 0.003 µm, respectively. Correspondingly, the specific surface area of RC, RMC-L and RMC-H was 811 ± 2.69 m^2^/kg, 1835.67 ± 24.03 m^2^/kg and 7917 ± 20.07 m^2^/kg, respectively (Figure 3C). It is likely that with the increase of MP concentration, more RMNPs pre-formed as nuclei in the solution, and a more homogeneous crystallization process of RH could be induced to form much smaller microcrystals. Unlike conventional drug loading strategies based on carrier materials, these carrier-free crystal formulations could achieve nearly 100% loading capacity (LC) and EE for the active component. As shown in Table 1, the LC and EE for ropivacaine in RMC-H was 88.17 ± 1.92% and 96.86 ± 1.99%, respectively, while those in RMC-L was 95.48 ± 1.15% and 99.36 ± 1.64%, respectively. As shown in Figure D and E, the FTIR spectra and XRD patterns of RMCs were quite different from those of RH but very similar to those of RC, suggesting that RMCs were similar to RC in component and detailed crystal structure.

Following these results, we compared the *in vitro* release profile of different formulations including RC, RH, RMNP-L, RMNP-H, RMC-L and RMC-H. As shown in Figure 3F, RH released more than 80% of the total drug content within the first 2 h and reached 100% release after 24 h, exhibiting a typical burst release. As shown in Figure S3, the release curve of RMNP-L and RMNP-H almost overlapped with that of free RH, suggesting that they were unable to slow down the release of ropivacaine. In contrast, all crystal formulations exhibited a typical slow-release profile, with RC, RMC-L and RMC-H reaching a cumulative release of 48.02 ± 24.24%, 62.26 ± 3.20% and 84.57 ± 2.63% respectively after 17 days. Furthermore, it was clear that with the crystal size getting smaller, the release rate became faster. It should also be noticed that RC exhibited an unstable release profile featured by very large variations, probably due to its highly inhomogeneous status. For this reason, the RC formulation was unsuitable for following studies *in vivo*.

Since both RMNP-L and RMNP-H released as quick as RH, in the pharmacokinetic study we only investigated the behaviors of the two RMC formulations and RH as the control. Specifically, 1% RH was used as a standard control since this is the maximum concentration available in clinic. As shown in Figure 3G and Table 2, 1%RH and 5%RH were quickly cleared from the blood. Due to the burst release and high concentration, the 5% RH formulation generated a very high maximum concentration (C_max_). In contrast, the circulation time of 5% RMC-L and 5% RMC-H was much longer. Furthermore, the C_max_ of 5%RMC-L and 5%RMC-H were much lower than that of 1% RH even though their drug content was 5 times higher, indicating their much lower risk of systemic toxicity. On the other hand, the area-under-the-curve (AUC) of 5% RMC-L, 5% RMC-H and 5% RH was 6171.9 ± 915.5, 7954.9 ± 425.9 and 8130.7 ± 1212.6 ng mL*h^-1^, respectively. Relatively lower AUC value of 5% RMC-L indicated an uncompleted drug release of this formulation, which was coincident with the release profile *in vitro*. On the other hand, RMC-H exhibited relatively longer T_1/2_ and T_max_ time, as well as a relatively higher C_max_ compared with RMC-L (Table 2), suggesting its more completed and sustained release.

### Prolonged anesthetic effect in SNB model

**Figure 4:**
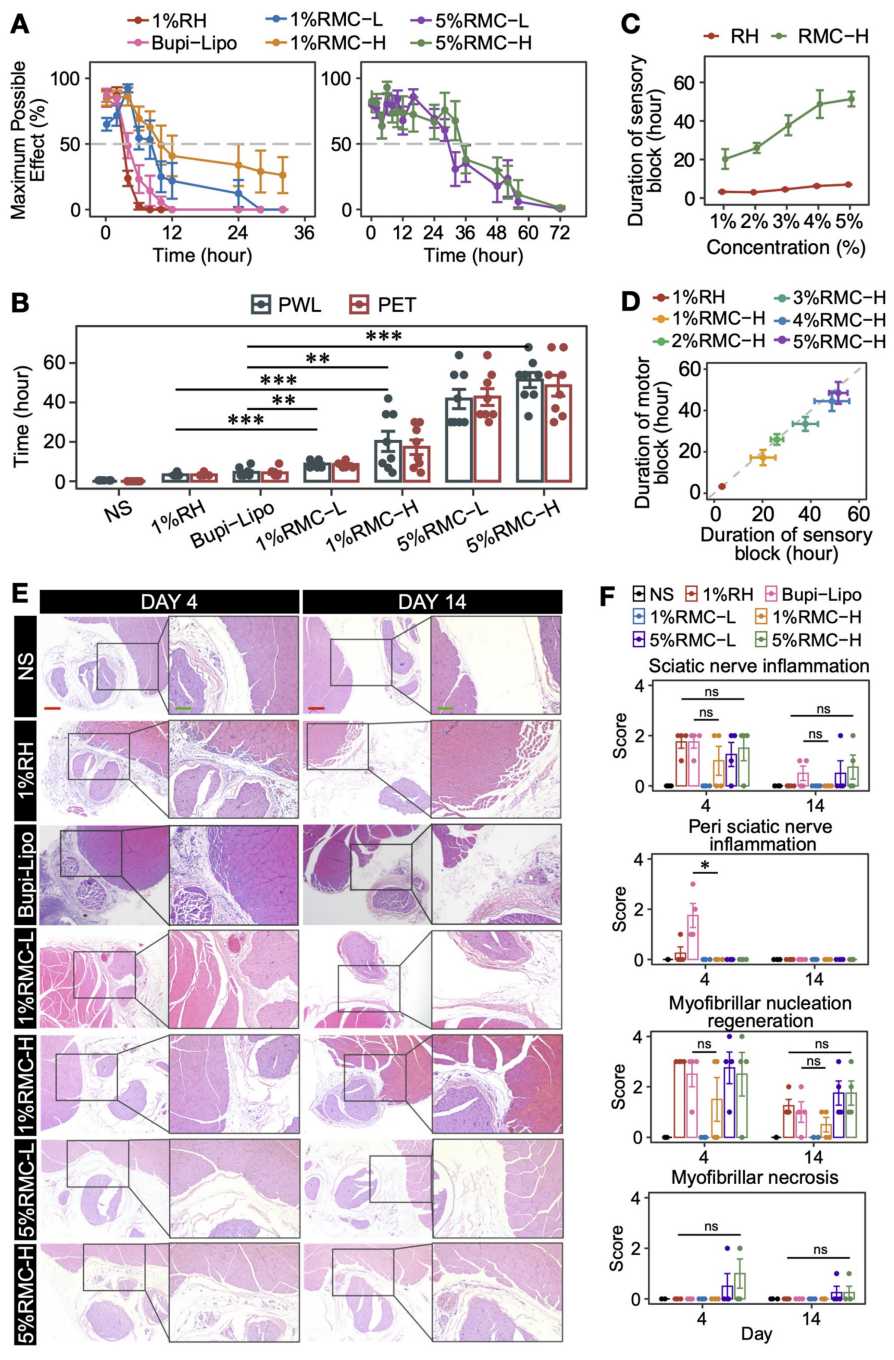
Nerve block efficacy of RH, Bupi-Lipo and RMC. (A) The MPEs for sensory block (PWL). (B) The duration of block time of different formulations, ***p < 0.001. (C) The sensory block duration of RH and RMC-H with different drug concentration. (D) Difference between the duration of sensory block and motor block in RH and RMC-H with different drug concentration. (E) Representative microscopic images of HE-stained tissue from different groups. Red scale bar = 200 μm; green scale bar = 100 μm. (F) Statistics analysis of pathology scores, the p-values were calculated by the Bonferroni correction, ns means no statistical difference, n = 4.

By using SNB model, we firstly investigated the nerve block efficacy of 1% RMC-L and 1% RMC-H and compared them with clinically available formulations, i.e., conventional 1% RH solution, as well as the long-acting Bupi-Lipo containing 1.33% bupivacaine. As shown in Figure 4A-B, 1 %RH formulation generated 3 h of sensory and motor block. Bupi-Lipo generated 5 h of block, which is only 2 h longer than 1%RH. On the other hand, the block time increased to 8.6 ± 1.7 h in the 1% RMC-L group and 20.3 ± 14.5 h in the 1% RMC-H group, respectively. Although the drug concentration of the RMC formulations was even lower than the Bupi-Lipo formulation, they achieved much longer block duration than the commercialized formulation, suggesting that RMC was a more effective slow-releasing system. Moreover, compared with 1% RMC-L, 1% RMC-H generated even longer block duration, which was coincident with the fact that RMC-H exhibited a more sustainable release profile and could retain an effective drug concentration for a longer time as revealed by the pharmacokinetic study.

By increasing the concentration of RMC formulations to 5%, even longer block durations could be achieved. In rats receiving 5% RMC-L, the duration of sensory block and motor block was 41.8 ± 13.9 and 42.8 ± 12.1 h, respectively. In rats receiving 5% RMC-H, the duration of sensory block and motor block was 51.4 ± 10.8 and 48.5 ± 14.9 h, respectively. Furthermore, the block duration could be deliberately controlled by adjusting the drug concentration of the RMC formulations. For example, by gradually increasing the drug concentration of the RMC-H formulation from 1% to 5%, sensory block duration ranging from 20.3 ± 14.5 to 51.4 ± 10.8 h could be easily achieved (Figure 4C). On the contrary, increasing the drug concentration of the RH formulation showed little effect on prolonging the block duration. It should also be noted that nearly all rats in the 5% RH group experienced obvious symptoms of systemic toxicity including trembling, vomiting and temporary unconsciousness, with 2 of 8 rats dead after injection, while neither 5% RMC-H nor 5% RMC-L formulation showed any sign of acute systemic or local toxicity. Furthermore, as shown in Figure 4D, for all RMC-H formulations with different drug concentration, the duration of sensory block was longer than motor block.

As shown in Figure 4E and 4F, mild local tissue damage including nerve inflammation and myofibrillar nucleation regeneration could be observed for all formulations containing ropivacaine, especially on day 4. In animals injected with 1% RH, sciatic nerve inflammation scores ranged from 0 to 3, which were significantly higher than those in the 1% RMC-L group (p = 0.0114), but showed no significant difference compared to the 1% RMC-H group (p = 0.32) or the 5% RMC-H group (p = 0.85). Similarly, no significant difference was observed for Bupi-Lipo compared to the 1% RMC-H group (p = 0.3173). However, Bupi-Lipo demonstrated a noticeable degree of perineural inflammatory infiltration around the sciatic nerve, whereas no such infiltration was observed in any of the RMC groups. When compared to the 1% RMC-H group, Bupi-Lipo exhibited a statistically significant difference in the perineural inflammation score (p = 0.0132). The inflammation in all groups almost completely disappeared on day 14, suggesting that the prolonged block duration of RMC formulations was not associated with elevated tissue damage, especially nerve damage.

### Protective anti-inflammatory efficacy and systemic safety evaluation of RMC

**Figure 5:**
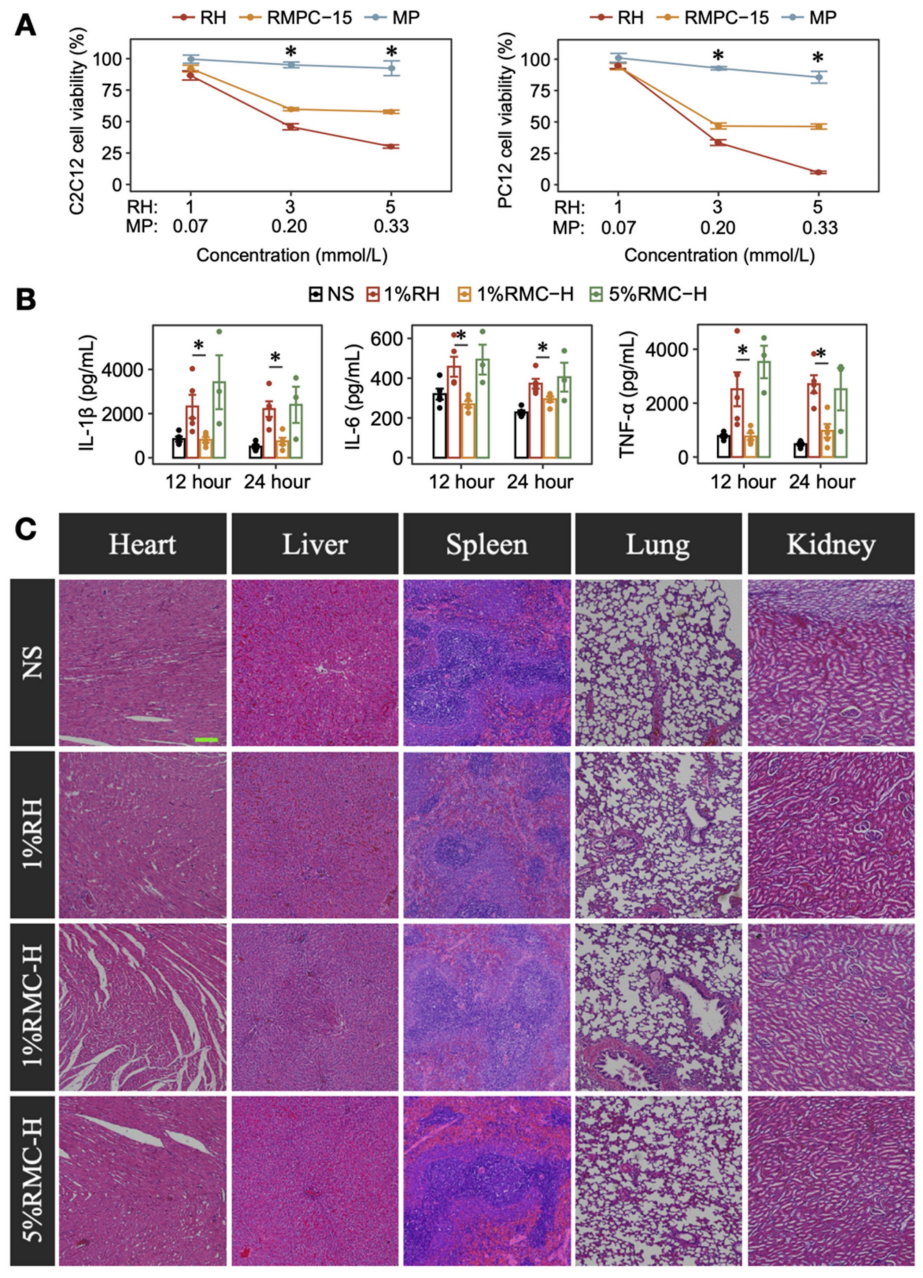
Anti-inflammatory effect and safety of RMC-H. (A) Comparison of cytotoxicity of RH and RMC in C2C12 and PC12 cells. The p-values were calculated by the Bonferroni correction, n = 4, * means p < 0.05 compared to the 1%RH group. (B) Concentrations of TNF-α, IL-1β, and IL-6 in the sciatic nerve and surrounding tissues of rats injected with RH or RMC. The p-values were calculated by the Bonferroni correction, n = 4, * p < 0.05. (C) Representative HE-stained microscopic images of heart, liver, spleen, lungs and kidneys 14 days after peri-sciatic administration of NS, 1%RH or different RMC formulations, scale bar = 200 µm.

C2C12 and PC12 cells were used to determine the potential protective effect of MP in the RMC-H formulation. As shown in Figure 5A, RH showed an obvious dose-dependent cytotoxicity on C2C12 and PC12 cells, while MP showed little effect on the cells' proliferation. Interestingly, for cells incubated with RMC-H formulations containing the same molar concentration of ropivacaine, cell viability was higher at the concentration of 3 mmol/L (p = 0.0209 for C2C12 cells, p = 0.0163 for PC12 cells) and 5 mmol/L (p = 0.0209 for C2C12 cells, p = 0.0090 for PC12 cells), suggesting the protective efficacy of MP in the RMC-H formulation.

Then we further evaluated the anti-inflammatory effect of MP in rat SNB model. As shown in Figure 5B, injection of 1% RH caused obvious local inflammation as characterized by the elevated expression of TNF-α, IL-1β and IL-6 compared with the NS group. In rat injected with 1% RMC-H, the expression of inflammatory factors was similar to that in the NS group, with a much lower expression of TNF-α (p = 0.0079 at 12 h and p = 0.011 at 24 h), IL-1β (p = 0.0079 at 12 h and p = 0.015 at 24 h) and IL-6 (p = 0.0119 at 12 h and p = 0.015 at 24 h) compared with the 1% RH group. Even in 5% RMC-H with much higher ropivacaine concentration, the level of inflammatory factors was similar to that of 1% RH (p = 0.65, p = 0.39 and p = 0.79 at 24 h for TNF-α, IL-1β and IL-6, respectively).

We also evaluated the effects of RMC on organ function, including liver and kidney function, through biochemical marker analysis. The results indicated that 5% RMC-H did not cause any increase in the assessed markers compared to 1% RH (Figure 5C). Some parameters showed higher values in the 1% RH group; however, these differences were not statistically significant, and the degree of increase did not reach clinical significance. Furthermore, none of the RMC-H formulations showed any injury in heart, liver, spleen, lung and kidney on day 14 (Fiugure 5D).

### Prolonged analgesic effect in rat postoperative pain model

**Figure 6:**
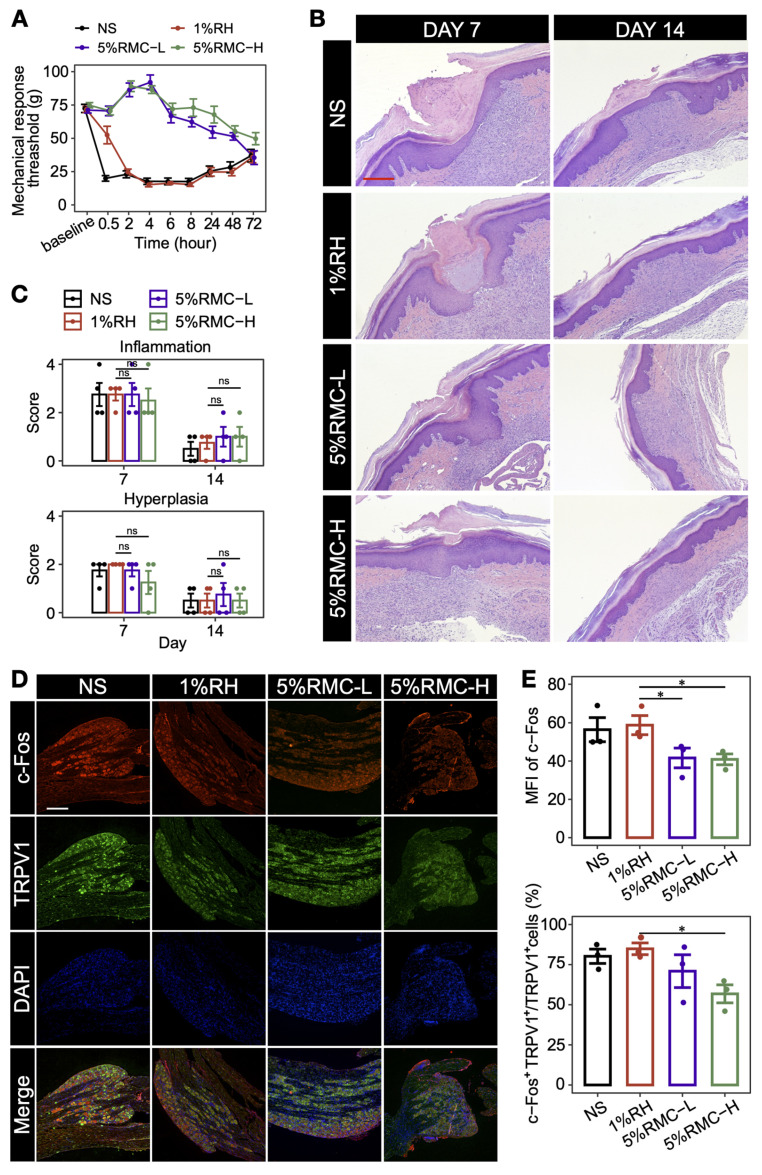
Long-acting analgesic effect of RMC in postoperative pain model. (A) MRT values of each group within 72 h; (B) Representative microscopic images of HE-stained tissue on day 7 and day 14. Scale bar = 100 μm. (C) The score of inflammation and hyperplasia analysis based on the tissue images. The p-values were calculated by the Bonferroni correction, ns means no statistical difference, n = 4. (D) representative immunofluorescence images for analyzing the expression of TRPV1 and c-Fos in L4 and L5 DRG (scale bar = 300 nm). (E) Statistical analysis of the MFI of c-Fos signal and the proportion of c-Fos^+^ neurons in the TRPV1^+^ neurons in different groups. The p-values were calculated by the Bonferroni correction, *p < 0.05 (n = 3).

To further evaluate the prolonged analgesic effect of 5% RMC formulations in the postoperative pain model, MRT was measured on rats injected with different formulations. As shown in Figure 6A, rats injected with NS exhibited an immediate and sharp drop of MRT to lower than 25 g, suggesting the occurrence of hyperalgesia after the surgery. For rats injected with 1% RH, the drug generated a transient analgesic effect, which diminished rapidly within 2 h. On the contrary, both 5% RMC formulations generated a significantly prolonged analgesic effect lasting for at least 48 h. Even after 72 h when the NS and 1% RH groups gradually recovered from hyperalgesia, the 5% RMC-H formulation still maintained a MRT higher than the two groups, suggesting that the formulation almost completely diminished postoperative pain caused by the surgery. At the meantime, there was no significant difference in inflammation response caused by the 5% RMC formulations compared with 1% RH (p = 0.98 on day 7; p = 0.86 on day 14), or even with the NS group (p = 0.92 on day 7; p = 0.73 on day 14). Hyperplasia scores showed similar results, with no significant differences in any of the four groups, indicating considerable safety of the 5% RMC formulations (Figure 6B-C).

It is known that the DRG plays an important role in the pathway of pain signal by receiving nociceptive stimulations from peripheral sensory nerve and transmitting them to the central nerve system 31. In the neurons in DRG, transient receptor potential cation channel, subfamily V, member 1 (TRPV1) is a key ion channel for the transmission of nociceptive stimulations, while the activation of TRPV1-positive neurons could be indicated by c-Fos staining. As shown in Figure 6D-E, both the MFI of c-Fos and the proportion of c-Fos positive neurons in TRPV1 neurons significantly decreased in the 5% RMC-H group compared with the NS group and 1% RH group, suggesting that the 5% RMC-H formulation has effectively suppressed the transmitting of pain signal from the incisional site to the DRG.

## Discussion

In many situations, pain is closely associated with inflammation, so anti-inflammatory drugs such as MP are conventionally combined with Las to manage both inflammation and pain 32, 33. For these simple combinations, it has been found that MP could slightly prolong the analgesic duration of LAs, presumably through the mechanisms of inhibiting the transmission of afferent c-fibers or immune modulation 34, 35. However, these pharmacodynamical functions actually have very limited efficacy in prolonging the analgesic duration. As noted in many studies, adding MP can only extend the duration of LAs from several hours to no more than 12 h 36.

In this study, we found that MP could self-assemble into nanoparticles, which could adsorb RH through electrostatic interaction, leading to the formation of RMNP nanoparticle complexes. This self-assembling system is somehow similar to a lidocaine-peptide complex system reported in our previous study 12, except that it was composed of pure drugs without involving in any materials. Molecule self-assembly has been well recognized as a novel strategy to develop drug delivery systems 37. Macromolecules such as polymers, peptides, nucleic acids and lipids have been extensively investigated as self-assembling biomaterials which can form carriers for drug delivery 38-41. More interestingly, recent studies have revealed pure drug-assembled nano-systems as a carrier-free drug delivery strategy, which have shown their intrinsic advantages including near 100% drug loading capacity, no material-related toxicity and low cost for preparation 42-45. To the best of our knowledge, this is the first time the self-assembling behavior of MP and its interaction with RH have been reported, which provided a potential strategy to adjust the release profile of ropivacaine by reshaping the drug formulation.

However, just forming the RMNP complex was not enough for slow-release as revealed in our study. As the second important step in the mechanism, transformation of freely-soluble RH to less soluble crystal form upon pH change turned out to be essential for improving release profile and prolonging anesthetic effect. In fact, it is already known that poorly-soluble LA crystals could be used as slow-releasing drug reservoir, and what matters is how to achieve a suitable release profile for maximized efficacy 12, 13, 27. In previous studies, plenty of materials were designed to disperse LA crystals into micro- to nano-particles, so that release rate could be controlled due to their different specific surface area 46-48. In this study, we could reach the same goal without involving any material. By changing the ratio of MP in the RMNP system, the particle size of microcrystals could be deliberately adjusted to generate an appropriate release speed for long-acting analgesia.

Since analgesia by nerve block will also inhibit motor function, long-term block duration may not always be the best choice in the clinic. In this regard, our formulation showed two potential advantages. On the one hand, drug concentration of the formulation could be deliberately adjusted to generate different durations of never block, which could provide suitable options to meet the requirement of different analgesic duration in different types of surgery. On the other hand, the slow-releasing formulation seemed to amplify the sensory-motor separation effect of ropivacaine, which further alleviate the side effect of motor block.

Except for extensively prolonged anesthetic duration, our system also greatly ameliorated the toxicity of ropivacaine through three mechanisms. Firstly, excellent slow-releasing profile of the formulation kept the local and plasma ropivacaine concentration within a relatively safe range, avoiding the risk of acute local tissue damage or systemic toxicity. Secondly, MP in the formulation also showed considerable anti-inflammation activity as shown in the cell and animal experiments, which further ameliorated local damage caused by ropivacaine. Lastly, this slow-release was based on the self-assemble nano-system of two clinically available drugs, avoiding potential toxicity associated with carrier materials.

To sum up, the RMC formulation developed in our study have some intrinsic advantages such as high drug loading capacity, low toxicity, long-lasting anesthetic duration and considerable safety. As an injectable formulation, its potential clinical application spans various scenarios such as nerve block, wound infiltration, transversus abdominis plane block and so on, significantly expanding the application of long-acting analgesia achieved with currently available formulations. However, the RMC formulation also has its own limitations and challenges still remain on its way to clinical application. Considering that MP is a bioactive steroid with certain adverse effects such as fluid and sodium retention, heightened susceptibility to infections, and an elevated risk of gastric ulceration, extra caution should be taken when RMC are used on patients susceptible to these adverse effects. For scaled-up production, optimization of its manufacturing parameters, including but not limited to rotational speed, concentration of pH adjusters, and the rate of their addition should be further exploited. Furthermore, additional investigations are required to establish the optimal storage conditions for the suspension and to comprehensively assess the content and stability of related substances. Despite these remaining challenges, RMC demonstrates significant potential and clinical value as a long-acting sustained-release formulation.

## Conclusion

In conclusion, MP as a dual-functional adjuvant could prolong the analgesic duration of RH pharmacokinetically and pharmacodynamically. Based on these self-assembly, crystallization and anti-inflammation mechanisms, we established a carrier-free slow-releasing platform for RH, which can extensively prolong the duration of anesthesia while exhibiting considerable safety comparable to current clinical formulations. Being only composed of clinically available drugs, this carrier-free formulation could be quickly evaluated and approved in clinic, indicating its promising translational future as a non-opioid formulation to treat postoperative pain. Considering the large number of other LAs and adjuvants sharing similar molecular property with ropivacaine and MP respectively, more long-acting LA formulations based on similar pure drug self-assembling system could be further exploited.

## Supplementary Material

Supplementary figures.

## Figures and Tables

**Table 1:** Loading capacity (LC) and encapsulation efficiency (EE) of RMNP and RMC

		LC	EE
RMNP-L	RH	98.19 ± 0.08%	12.64 ± 0.26%
	MP	1.72 ± 0.08%	23.56 ± 3.91%
RMNP-H	RH	93.34 ± 0.1%	23.39 ± 0.43%
	MP	6.40 ± 0.09%	16.22 ± 1.42%
RMC-L	RH	95.48 ± 1.15%	99.36 ± 1.64%
	MP	1.26 ± 0.02%	80.75 ± 3.04%
RMC-H	RH	88.17 ± 1.92%	96.86 ± 1.99%
	MP	7.01 ± 0.18%	55.64 ± 6.16%

**Table 2:** The pharmacokinetic parameters of different formulations

		1%RH	5%RH	5%RMC-L	5%RMC-H
AUC_0-t_	ng/(mL*h)	1757.1 ± 246.2	8130.7 ± 1212.6	6171.9 ± 915.5	7954.9 ± 425.9
AUC_0-∞_	ng/(mL*h)	1769.2 ± 249.3	8463.7 ± 1118.2	6840.6 ± 699.1	9005.7 ± 524.1
T_1/2_	h	1.1 ± 0.1	1.8 ± 0.4	8.0 ± 4.4*	10.7 ± 7.7*
T_max_	h	1.1 ± 0.4	0.7 ± 0.4	9.2 ± 2.9*	11.5 ± 6.6*
C_max_	ng/mL	673.9 ± 125.3	2216.6 ± 411.4	207.6 ± 36.6*	269.3 ± 56*

* : Means statistically significant difference compared to the 1% RH group.
