# Supplementary material for: A carrier-free long-acting ropivacaine formulation using methylprednisolone sodium succinate as a dual-functional adjuvant
Source: Theranostics. 2025 Mar 3;15(9):3862–76. doi: 10.7150/thno.107397 (PMC11980666; doi:10.7150/thno.107397)
Supplement: Supplementary file 1 — Supplementary figures. [file thnov15p3862s1.pdf]

A carrier-free long-acting ropivacaine formulation using methylprednisolone sodium succinate as a dual-functional adjuvant

Weiwei Wu[1,3#], Yan Wang[2#], Feng Qiu[1,3], Yingxian Dong[2], Jing Liu[1,3], Yujun Zhang[1,3], Deying Gong[3], Yi Kang[3], Guoyan Zhao[3], Congyan Liu[3], Yuncheng Li[3], Tao Zhu[1,4*], Wensheng Zhang[1,3*], Guowei Che[2,5*]

[1] Department of Anaesthesiology, West China Hospital, Sichuan University, Chengdu, China

[2] Department of Thoracic Surgery, West China Hospital, Sichuan University, Chengdu, China

[3] Laboratory of Anaesthesia and Critical Care Medicine, National-Local Joint Engineering Research Centre of Translational Medicine of Anaesthesiology, West China Hospital, Sichuan university, Chengdu, China

[4] Research Unit for Perioperative Stress Assessment and Clinical Decision, Chinese Academy of Medical Sciences (2018RU012), West China Hospital, Sichuan University, Chengdu, China

[5] Lung Cancer Center, West China Hospital, Sichuan University, Chengdu, China

Supplementary Figures

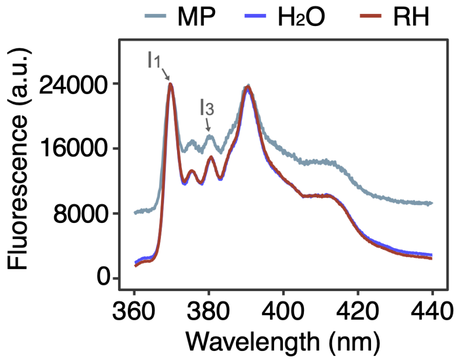

Figure S1. Comparison of pyrene fluorescence spectra in 4 mg/mL MP, 40 mg/mL RH and H_2O. With the I1 peak normalized, increased I3 peak in MP indicated strong hydrophobic interaction and suggested the self-assembly behavior. On the contrary, I3 peak in RH and H_2O completely overlapped, suggesting the absence of hydrophobic interaction in RH solution, which means RH could not undergo self-assembly.

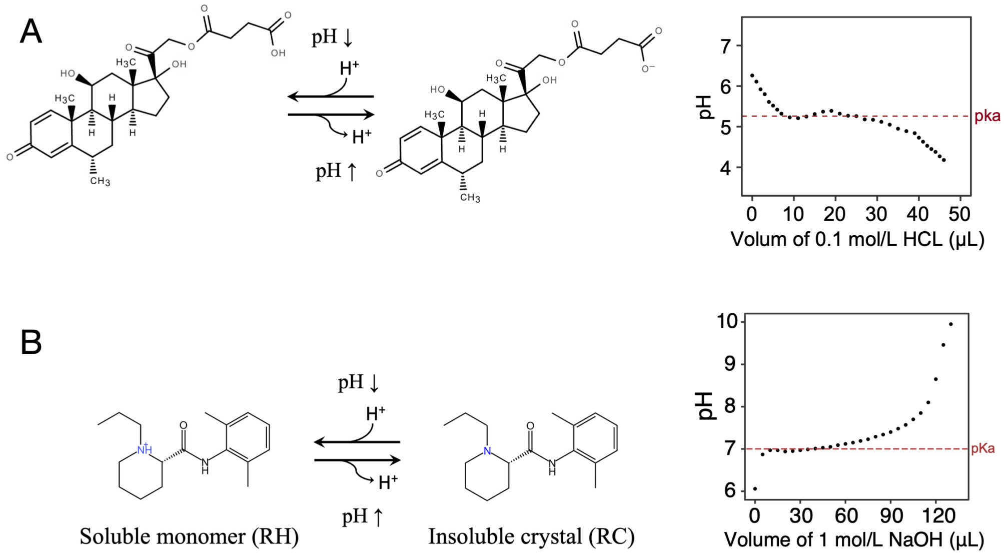

Figure S2. (A) The dissociation equilibrium of MP and the pKa determined by HCl titration; (B) The dissociation equilibrium of RH and the pKa determined by NaOH titration.

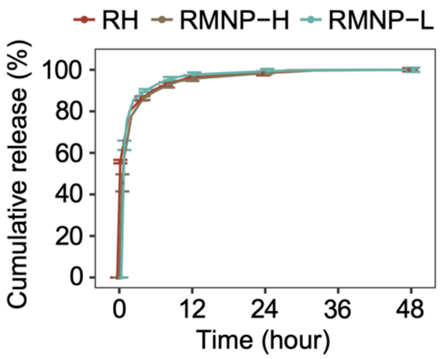

Figure S3. The *in vitro* release curves of RH, RMNP-L and RMNP-H (n=3). All formulations exhibited a similar burst-release profile.
